# Structural basis of stereospecific reduction by quinuclidinone reductase

**DOI:** 10.1186/2191-0855-4-6

**Published:** 2014-02-07

**Authors:** Daijiro Takeshita, Michihiko Kataoka, Takuya Miyakawa, Ken-ichi Miyazono, Shoko Kumashiro, Takahiro Nagai, Nobuyuki Urano, Atsuko Uzura, Koji Nagata, Sakayu Shimizu, Masaru Tanokura

**Affiliations:** 1 Department of Applied Biological Chemistry, Graduate School of Agricultural and Life Sciences, University of Tokyo, 1-1-1 Yayoi, Bunkyo-ku, Tokyo 113-8657, Japan; 2 Division of Applied Life Sciences, Graduate School of Agriculture, Kyoto University, Kitashirakawa-Oiwakecho, Sakyo-ku, Kyoto 606-8502, Japan; 3 Division of Applied Life Sciences, Graduate School of Life and Environmental Sciences, Osaka Prefecture University, 1-1 Gakuencho, Naka-ku, Sakai, Osaka 599-8531, Japan; 4 Research and Development Center, Nagase & Co., Ltd., 2-2-3 Murotani, Nishi-ku, Kobe 651-2241, Japan; 5 Faculty of Bioenvironmental Science, Kyoto Gakuen University, Sogabe-cho, Kameoka 621-8555, Japan

**Keywords:** Crystal structure, NADPH-dependent enzyme, Asymmetric reduction, 3-Quinuclidinone reductase, (*R*)-3-quinuclidinol

## Abstract

Chiral molecule (*R*)-3-quinuclidinol, a valuable compound for the production of various pharmaceuticals, is efficiently synthesized from 3-quinuclidinone by using NADPH-dependent 3-quinuclidinone reductase (RrQR) from *Rhodotorula rubra*. Here, we report the crystal structure of RrQR and the structure-based mutational analysis. The enzyme forms a tetramer, in which the core of each protomer exhibits the α/β Rossmann fold and contains one molecule of NADPH, whereas the characteristic substructures of a small lobe and a variable loop are localized around the substrate-binding site. Modeling and mutation analyses of the catalytic site indicated that the hydrophobicity of two residues, I167 and F212, determines the substrate-binding orientation as well as the substrate-binding affinity. Our results revealed that the characteristic substrate-binding pocket composed of hydrophobic amino acid residues ensures substrate docking for the stereospecific reaction of RrQR in spite of its loose interaction with the substrate.

## Introduction

The chirality of chemical compounds is an important property for their availability as building blocks for pharmaceuticals and chemicals, including chemical catalysts, liquid crystals, flavors, agrochemicals, and medicines. As is often the case, only one optical isomer primarily or completely exhibits the intended activity. Therefore, it is necessary to develop methods to produce optically pure compounds for use in the industrial production of chiral compounds. Biotransformations using whole cells and enzyme-catalyzed reactions have been attempted for the production of various chiral compounds. The enzymatic asymmetric reduction of prochiral carbonyl compounds is a promising method for the production of chiral alcohols, because enzymes have certain advantages for use in chemical reactions under mild conditions and with chemo-, regio-, and stereo-selectivity (Hummel 1997; Kataoka et al. 2003; Goldberg et al. 2007). Structural information about useful enzymes would enable the generation of new enzymes exhibiting high activity, enhanced stability, and altered-coenzyme specificity, and would also enable elucidation of the detailed reaction mechanisms and substrate-binding specificities.

One of the chiral alcohols, (*R*)-3-quinuclidinol, is a useful compound applicable to the synthesis of various pharmaceuticals, such as talsaclidine and revatropate (Rzeszotarski et al. 1988; Bietti et al. 1990; Cross and Stobie 1993; Takeuchi et al. 1996; Alabaster 1997; Ward et al. 1998; Leusch et al. 2000; Ishihara et al. 2004). So far, chemical and biochemical methods have been applied to produce (*R*)-3-quinuclidinol (Stembach and Kaiser 1952; Rehavi et al. 1977; Muchmore 1993; Bossard 1997; Brieden 1998; Matsuyama and Hamatani 1998; Sato and Enomoto 1999; Nomoto et al. 2003; Yamamoto et al. 2003; Takenaka 2006), and most of these methods take the approach of resolving a racemic mixture. Recently, for the production of (*R*)-3-quinuclidinol, 3-quinuclidinone reductase (RrQR) was isolated from *Rhodotorula rubra* (Uzura et al. 2009). The purified enzyme from *R. rubra* is specific to NADPH as a coenzyme and stereospecifically produces the (*R*)-enantiomer of 3-quinuclidinol from 3-quinuclidinone. Furthermore, a coexpression system for the 3-quinuclidinone reductase and glucose dehydrogenase using *Escherichia coli* as a host was developed for the large-scale production of (*R*)-3-quinuclidinol. The system using the enzyme is superior to former kinetic resolution methods in that it has a higher molar yield, requires no substrate modification, and so on (Uzura et al. 2009).

Based on the amino acid similarity, RrQR belongs to the short-chain dehydrogenase/reductase family. Though the proteins in this family have low sequence identities, they share a similar α/β Rossmann-fold core. In contrast to the fold similarity, oxidoreductases, which make up the majority of the family, exhibit various substrate specificities (Oppermann et al. 2003), indicating that the mechanisms to accommodate structurally different substrates are diverse. The molecular identification and characterization of the enzymes of this family, which have diverse activities capable of producing useful molecules, would be helpful for the utilization of these enzymes. To elucidate the detailed molecular mechanism underlying the stereospecific reduction of the 3-quinuclidinone, we determined the crystal structure of RrQR in complex with NADPH. Modeling and mutation analyses of RrQR indicated a novel mechanism underlying the stereospecific reaction led by a characteristic interaction, explaining how optically pure (*R*)-3-quinuclidinol is synthesized from 3-quinuclidinone.

## Materials and methods

### Crystallization and structure determination of RrQR

RrQR was expressed, purified, and crystallized as described previously (Takeshita et al. 2009). Briefly, the crystals were grown at 278 K using the sitting-drop vapor-diffusion method with a reservoir solution containing 100 mM CHES (pH 10.0), 30% PEG8000, and 3% sucrose. The X-ray diffraction data set was collected at 95 K at Beamline BL-5A of the Photon Factory, KEK (Tsukuba, Japan). The data were processed using HKL2000 (Otwinowski and Minor 1997). The crystals belonged to space group *P*4_1_2_1_2 with unit-cell parameters of *a* = *b* = 91.3 Å and *c* = 265.4 Å. The data-collection statistics of the RrQR crystal are summarized in Table 1 and Additional file 1: Table S1. The reason for the lower completeness in the highest resolution shell is the limitation of the camera size at the beamline. The reflection data to 2.2 Å resolution were used for the structural analysis because the values of *R*_merge_, redundancy and completeness in the highest resolution shell are valuable for the refinement. The asymmetric unit contained four molecules of RrQR and the RrQR structure was solved by molecular replacement using Phaser (McCoy et al. 2007) with *Thermotoga maritima* TM0441 (PDB ID 1vl8) as a search model. The model was refined using REFMAC5 (Murshudov et al. 1997) and CNS (Brünger et al. 1998). The model was manually rebuilt using XtalView (McRee 1999). The refined structure was assessed using PROCHECK (Laskowski et al. 1993). The figures were prepared with PyMOL (DeLano 2002). Energy minimization of the substrate-binding model was performed with GROMACS-4.6.3 (Hess et al. 2008) according to online tutorials (http://www.gromacs.org/).

**Table 1:** Data collection and refinement statistics of the RrQR crystal

**Data collection**	
X-ray source	PF BL-5A
Wavelength (Å)	1.0000
Space group	*P*4_1_2_1_2
Unit-cell parameters (Å)	*a* = *b* = 91.3, *c* = 265.4
Resolution (Å)^a^	20.0-2.20 (2.28-2.20)
Observed reflections	689047
Unique reflections	55949
Completeness (%)^a^	96.5 (81.0)
*R*_*merge*_ (%)^a,b^	9.2 (23.9)
<I>/<σ(I) > ^a^	55.6 (11.4)
**Refinement**	
Resolution range (Å)	20.0-2.20
R_factor_(%)^c^	19.2
R_free_(%)^d^	23.0
Protein atoms	8020
NADPH atoms	192
Water molecules	302
r.m.s deviaton from ideal	
Bond lengths (Å)	0.011
Bond angles (deg.)	1.43
Ramachandran plot^e^	
Most favored regions (%)	91.7
Allowed regions (%)	8.3

^a^Data of the highest-resolution shell are shown in parentheses.

^b^*R*_merge_ = ∑  _*hkl*_ ∑ _*i*_|*I*_*i*_(*hkl*) − < *I*(*hkl*) > |/∑_*hkl*_  ∑ _*i*_ I_*i*_(*hkl*), where *I*_*i*_(*hkl*) is the *i*-th intensity measurement of reflection *hkl*, including symmetry-related reflections, and < *I*(*hkl*) > is its average.

^c^*R*_*factor*_ = ∑ _*hkl*_|F_obs_(*hkl*) − F_calc_(*hkl*)|/∑_*hkl*_|F_obs_(*hkl*)|.

^d^*R*_free_ = the cross-validation *R*_factor_ for 5% of reflections that were not used in refinement.

^e^Ramachandran plot assessment using PROCHECK (Laskowski et al. 1993).

### 3-Quinuclidinone reductase assay of wild-type and mutant RrQR enzymes

The reaction mixture comprised, in 2 ml, 500 mM 3-quinuclidinone · HCl, 566 mM glucose, 1.2 mg/ml NAD^+^ or NADP^+^, 0.4 mg/ml glucose dehydrogenase (Amano Enzymes, Nagoya, Japan), 200 mM potassium phosphate buffer (pH 7.0), and an appropriate amount of purified enzyme. After 20 h of incubation at 30°C, the reaction was terminated by addition of an equal volume of saturated Na_2_CO_3_, and then the formed 3-quinuclidinol was extracted with an equal volume of chloroform. The optical purity of 3-quinuclidinol was determined by gas-liquid chromatography analysis, as described previously (Uzura et al., 2009).

Mutations were introduced into the plasmid pET28a containing the RrQR gene. The mutant proteins were purified using a Ni-NTA column (Qiagen). The activities of the wild-type and mutant RrQR enzymes were analyzed as follows. The standard assay mixture was comprised of 2.5 ml, 125 mM 3-quinuclidinone-HCl, 350 μM NADPH, and 200 mM potassium phosphate buffer (pH 7.0), and the appropriate amount of the purified enzyme. After 2 min of incubation without the enzyme at 37°C, the reaction was started by adding the enzyme, and then the decrease in absorbance at 340 nm due to NADPH oxidation was determined. One unit of RrQR activity was defined as the amount catalyzing the oxidation of 1 μmol NADPH per min.

### Accession number

Coordinates and structure factors have been deposited in the Protein Data Bank with accession number 4O0L.

## Results

### Overall structure

The crystal structure of RrQR was determined at 2.2-Å resolution and refined to a crystallographic *R*-factor of 0.192 and *R*_free_ of 0.230 (Table 1 and Additional file 1: Table S1). The asymmetric unit of the crystal contains four protomers of RrQR. The final model includes residues 7‒272 for each protomer. There is no electron density for N-terminus residues 1‒6 in any of the four protomers, indicating that the region is flexible. The four protomers in the asymmetric unit are very similar to one another, with root-mean-square (r.m.s.) deviations of 0.20‒0.24 Å. A clear electron density for NADPH is observed in each protomer, and the structures of the four NADPH molecules in the asymmetric unit exhibit similar conformations.

**Figure 1:**
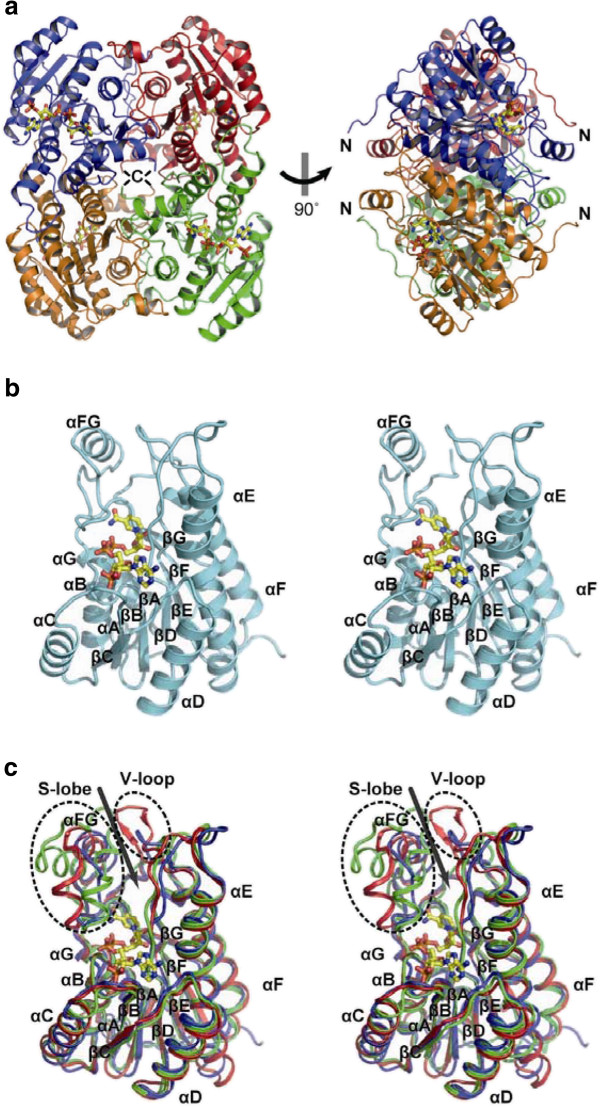
**Overall structure of RrQR. a** Overall view of RrQR. The protomers are shown in blue, red, orange, and green; NADPH molecules are yellow (left). Side view of RrQR (right). **b** Stereoview of the structure of the RrQR protomer, presented in light blue. The NADPH molecule is shown as a yellow stick model. **c** Stereoview of the protomer structure of RrQR, and superimposition with similar structures. RrQR, mannitol 2-dehydrogenase (PDB ID: 1h5q), and trihydroxynaphthalene reductase (PDB ID: 1g0n) are shown in blue, red, and green, respectively. The NADPH molecule of RrQR is shown as a yellow stick model. The α-helices, αA-αG, and the β-strands, βA-βG, are labeled. The αFG between αF and αG is also labeled. The small lobe and variable loop are encircled by dotted lines. The deep cleft is shown by a gray arrow.

The crystal structure of RrQR shows a tightly associated homotetramer (Figure 1a). The tetrameric structure is consistent with the results of a previous gel-filtration chromatographic analysis (Uzura et al. 2009). Like other SDR family proteins, the RrQR protomer can be divided into two domains: the core and small lobe structures (Figure 1b and c). The core structure exhibits the α/β Rossmann fold, with seven α-helices, two 3_10_-helices and a central parallel β-sheet consisting of seven strands. The small lobe is away from the core, which lies between βF and αG (residues 212-238) and contains one helix αFG. A deep cleft exists between the core and the small lobe, and reaches to about the middle of the molecule. The NADPH cofactor is located at the bottom of the cleft. A remarkably extended structure is found in the N-terminal region, consisting of residues 7‒19. There is a hydrophobic stacking of P10 with Y229 on αFG of the neighboring protomer, which would contribute to the quaternary structural stability (Additional file 1: Figure S1).

The search for structurally homologous proteins was performed with the Dali server, and revealed several similar proteins: β-ketoacyl acyl carrier protein reductase (r.m.s. deviation of 1.2 Å for 229 Cα atoms (Fisher et al. 2000; PDB ID: 1edo)), trihydroxynaphthalene reductase (r.m.s. deviation of 1.2 Å for 219 Cα atoms (Liao et al. 2001; PDB ID: 1g0n)), and mannitol 2-dehydrogenase (r.m.s. deviation of 1.3 Å for 234 Cα atoms (Hörer et al. 2001; PDB ID: 1h5q)). Structural comparison showed that their core structures are almost superimposable, whereas remarkable structural diversities exist around the cleft (Figure 1c). The cleft is formed by the small lobe and a loop on the side opposite the lobe; this loop lies between βE and αF (residues 171-178). In the small lobe (S-lobe), RrQR has one helix αFG, whereas the other enzymes each have two helices. Upon the loop, the mannitol 2-dehydrogenase has a small antiparallel β-sheet, and the other enzymes have different structures. Thus, we named the loop the variable loop (V-loop) (Figure 1c). The deep cleft is located above the B-face of the nicotinamide ring, and it has been described as the substrate-binding site in SDR family proteins. Therefore, the diverse structures around the cleft could enable the family proteins to accommodate various substrates.

### NADPH-binding mode of RrQR

**Figure 2:**
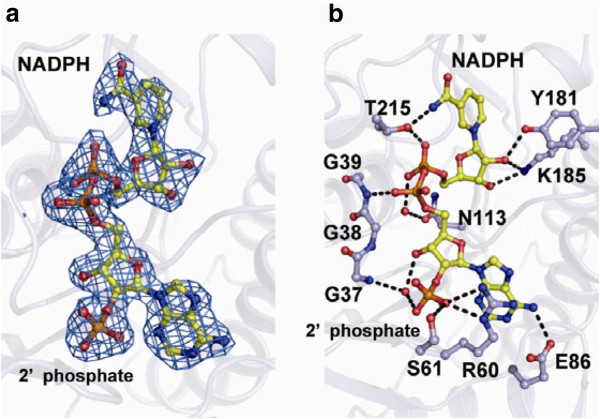
**The NADPH-binding site of RrQR. a** The NADPH molecule is represented by a stick model, and the NADPH omit *F*_O_-*F*_C_ map contoured at 3.0 σ is shown in light blue. There is no cutoff of the radius of the atoms for the display of the electron density. **b** Side chains of the amino acid residues forming hydrogen bond(s) with the NADPH molecule are represented by a stick model, and the hydrogen bonds are shown by dotted lines. Residues G37 through G39 are also shown by a stick model.

The bound NADPH is in an extended conformation, and the nicotinamide and adenine rings are about 10.5 Å apart (Figure 2). Both ribose rings are in the C2′-endo conformation. The adenine ring is in the *anti* conformation, and the nicotinamide ring is in the *syn* conformation. The conformation and the space above the nicotinamide ring allow the 4-*pro-S* hydride transfer from the B-side of the nicotinamide ring. The enzyme interacts with the NADPH through hydrogen bonds (Additional file 1: Figure S2). Side chains of the residues R60, S61, E86, Y181, K185, and T215 form hydrogen bonds with the NADPH molecule, and a side chain of N113 forms a hydrogen bond with a water molecule that is hydrogen-bonded to the NADPH (Figure 2 and Additional file 1: Figure S2). Among these residues, R60 and S61 form hydrogen bonds with the 2′-phosphate group of the NADPH; these bonds are involved in the discrimination of NADPH from NADH. The main chains of the other residues, G35, G37, G39, G41, L58, A62, V87, T165, P210, and G211, also form hydrogen bonds with the NADPH molecule.

### Structure of the active site

**Figure 3:**
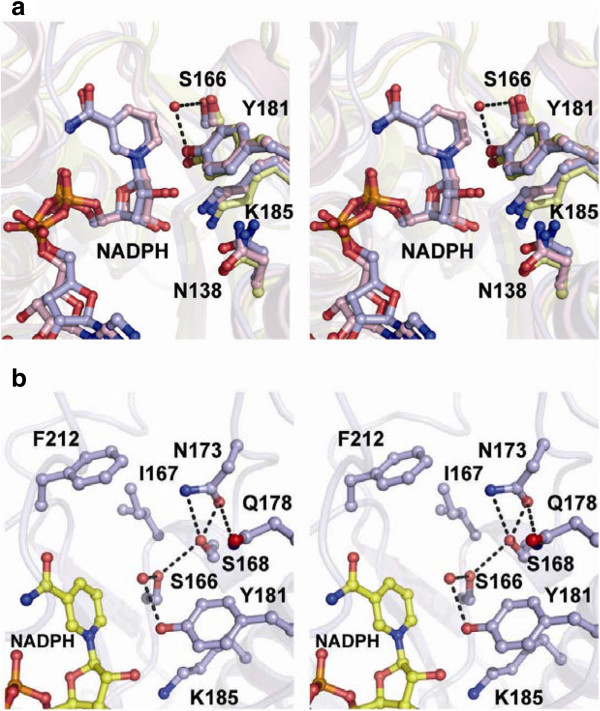
**The active site of RrQR. a** Stereo view of the active site of RrQR and its superimposition with those of TR-II and 3β/17β-hydroxysteroid dehydrogenase. The catalytic residues, serine, asparagine, tyrosine, and lysine, are shown by a stick model. RrQR, TR-II, and 3β/17β-hydroxysteroid dehydrogenase are shown in light blue, pink, and pale yellow, respectively. The residues of RrQR are labeled. **b** Stereo view of the substrate-binding site of RrQR. A water molecule in the active site is shown as a sphere, and hydrogen bonds are shown by dotted lines. The hydrophobic wall is comprised of I167 and F212.

The active site of SDR enzymes is composed of a triad of catalytically important residues: serine, tyrosine, and lysine (Jörnvall et al. 1995). More recently, an asparagine residue or a histidine residue at the corresponding position was added to the catalytic triad (Filling et al. 2002; Kubota et al. 2011), since a water molecule bound to the main-chain carbonyl group of the asparagine or histidine is assumed to participate in the reductive reaction. The configuration of these catalytic tetrad residues is well superimposed on that of RrQR (Figure 3a), and thus the catalytic reaction would be achieved through the same proton relay (Filling et al. 2002).

To identify the catalytic site where the carbonyl group of 3-quinuclidinone is converted to the hydroxyl group, we performed a mutation analysis of RrQR. In this reductase, residues N138, S166, Y181, and K185 correspond to the catalytic tetrad. Mutations of these residues, S166A, Y181A, Y181F, and K185A, resulted in a complete loss of activity (Table 2). The results reveal the catalytic residues of RrQR, providing a basis for the reaction-based model as described below.

**Table 2:** Quinuclidinone reductase activities of the wild-type and mutant RrQR enzymes

	**Mutation**	**Relative activity for 3-qinuclidinone**^**a**^	***K***_**m**_**(mM)**^**b**^	***V***_**max**_**(unit/mg)**^**a**^	***k***_**cat**_**(sec**^**-1**^**)**
	WT	100	440	31.2	15.1
Catalytic site	S166A	ND			
	Y181A	ND			
	Y181F	ND			
	K185A	ND			
Substrate-binding site	I167A	ND			
	I167V	24.5	308	5.56	2.69
	S168A	115.7	168	19.9	9.64
	N173A	97.8	100	11.6	5.62
	Q178A	164.5	103	20.2	9.79
	F212A	ND			
	F212L	ND			

ND: not detected.

^a^The analyses were performed in the presence of 125 mM 3-quinuclidinone.

^b^The analyses were performed in the presence of 350 μM NADPH.

### Substrate-binding site and 3-quinuclidinone-binding model

A small space is found above the B-face of the nicotinamide ring, and is assumed to be the substrate-binding site. This small pocket, with a diameter of 7 Å and a depth of 8 Å, is formed by S166, I167, S168, N173, Q178, Y181, and F212 (Figure 3b). None of these residues is charged, and the most notable characteristic of the pocket is a hydrophobic wall composed of I167 and F212, located at about the bottom of the pocket.

**Figure 4:**
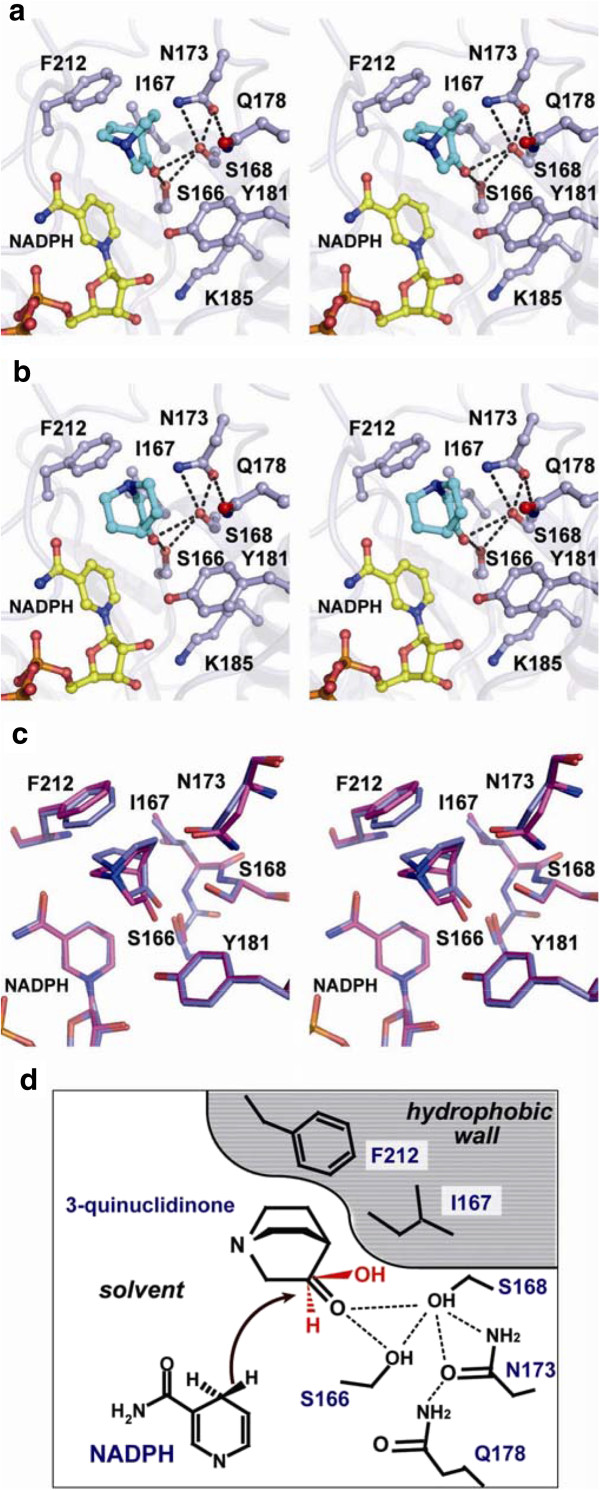
**Stereospecific binding for the asymmetric reduction of 3-quinuclidinone by RrQR.** A 3-quinuclidinone in the active site of RrQR is shown by a stick model and in cyan. **a** The catalytic model of 3-quinuclidinone-RrQR ensures the product (*R*)-3-quinuclidinol. The hydrophobic wall comprised of I167 and F212 is adjacent to the hydrophobic side of the substrate, which stabilizes the substrate binding. The tertiary amine group of the substrate is exposed to solvent. **b** The noncatalytic model of 3-quinuclidinone-RrQR leads to the product (*S*)-3-quinuclidinol. The hydrophobic wall is adjacent to the amine group of the substrate, resulting in an unstable state. A hydrophobic surface of the substrate is exposed to solvent. **c** The refined structure of RrQR by an energy minimization (red) is superimposed on the structure of the catalytic model shown in Figure 4**a** (blue). **d** Schematic diagram of the catalytic mechanism of RrQR. The hydride transfer from NADPH to the carbonyl carbon of 3-quinuclidinone is depicted by an arrow. The hydrophobic wall comprised of I167 and F212 is adjacent to the hydrophobic surface of the substrate. The hydrogen bonds in the active site are shown by dotted lines. The hydroxyl group of the product is shown in red.

To obtain further insights into the mechanism underlying the RrQR reaction, we built a substrate-binding model that ensures a stereospecific reaction (Figure 4). Structural analyses of TR-II, a member of the SDR family, revealed that the catalytic reaction is achieved through a slight rotational movement of the substrate molecule (Yamashita et al. 1999; Yamashita et al. 2003). The chemical reaction catalyzed by the enzymes TR-II and RrQR is the same, and involves the reduction of a carbonyl group to a hydroxyl group. Superimposition of their structures shows that the configurations of the catalytic residues (S166, Y181, and K185) and the nicotinamide ring of NADPH are essentially identical (Figure 3a). This structural similarity leads to the rational substrate-binding model of RrQR based on the complex structure of the TR-II-substrate complex.

The ketone group of the substrates has a trigonal planar configuration, with C-C-O and C-C-C bond angles of about 120˚. Therefore, the superimposition of the ketone group (C-CO-C) of 3-quinuclidinone to that of tropinone can be limited to two patterns, which are related by a 180° rotation about the carbonyl C-O bond axis (Figure 4a and b). The reaction is achieved through the proton transfer to the carbonyl group of a substrate from the catalytic residue tyrosine, and through the nucleophilic attack of the hydride of NADPH on the carbonyl carbon of the substrate. Thereby, the hydroxyl group of the product relocates to the side opposite that of the nicotinamide ring. The geometry presented in Figure 4a agrees with the catalytic reaction of RrQR, producing (*R*)-3-quinuclidinol from 3-quinuclidinone. On the other hand, the geometry in Figure 4b results in the production of another stereoisomer, (*S*)-3-quinuclidinol, which does not agree with the catalytic reaction of RrQR. The enzyme RrQR produces only (*R*)-3-quinuclidinol. Thus we describe the geometry in Figure 4a as a catalytic model and that in Figure 4b as a noncatalytic model.

We then evaluated the surroundings of the active site for the two models. The molecule 3-quinuclidinone contains a tertiary amine group, which has basic and hydrophilic properties due to a lone electron pair. In the catalytic model, the amine group is exposed to solvent, while on the opposite side, hydrophobic hydrocarbons of 3-quinuclidionone are buried in the small pocket and adjacent to the hydrophobic wall composed of I167 and F212, resulting in a stable state. On the other hand, in the noncatalytic model, the amine group is located adjacent to a hydrophobic wall composed of I167 and F212, and the hydrocarbons of 3-quinuclidinone are exposed to the solvent, thereby resulting in an unstable state. The differences between the two models can explain how the RrQR enzyme produces (*R*)-3-quinuclidinol prior to (*S*)-3-quinuclidinol from 3-quinuclidinone.

### Critical residues in the substrate-binding site

The purified RrQR enzyme catalyzed the production of (*R*)-quinuclidinol from 3-quinuclidinone, with an enantiomeric excess of >99.9% (Uzura et al. 2009). The residues interacting with the substrate were predicted by the program Ligplot (Laskowski and Swindells 2011, Additional file 1: Figure S3), and Table 2 summarizes the results of the mutation analysis of the substrate-binding site. The analysis revealed that alanine mutations at hydrophobic residues I167 and F212 significantly affect the activity. The F212A and F212L mutants completely lost their catalytic activity, indicating the importance of the bulky hydrophobic property of phenylalanine. The accessible surface areas of phenylalanine and leucine are 30.9 Å^2^ and 26.9 Å^2^, respectively. Therefore, the bulky hydrophobic surface generated from the additional hydrocarbons plays a significant role in the activity. Similarly, the I167A mutant lost the activity, and the I167V mutant showed a significant decrease in the activity, to 24.5% of the wild type. With respect to the catalytic model presented in Figure 4a, the hydrocarbons of I167 and F212 would have direct hydrophobic interactions with the substrate, because the distances between the substrate and the side chains of I167 or F212 are within 4 Å.

In contrast to the mutations at the hydrophobic residues I167 and F212, the mutations at polar amino acid residues S168, N173 and Q178 did not exhibit significantly reduced activity (Table 2). In these mutations, the *K*_m_ values are decreased and the *k*_cat_ values are decreased, meaning that the affinities of the mutants are increased but the rates of the catalysis are decreased. The higher affinities of S168A, N173A and Q178A may be due to the increase of hydrophobicity favorable for the substrate binding. The reductions of the *k*_cat_ value of these mutants suggest that the hydrogen network including catalytic residue S166 may be favorable for the reaction (Figure 4).

We subsequently refined the structure of the substrate-binding model by means of energy minimization, indicating that slight movements of residues I167, N173 and F212 enable the enzyme to adapt to 3-quinuclidinone (Figure 4c). Therefore, the active site of the enzyme has a preference for the substrate without large structural rearrangements.

The residues forming the substrate-binding pocket are located in the small lobe (residue F212) and on or near the variable loop (residues I167, S168, N173, and Q178). The two structural elements, the small lobe and the V-loop, are characteristic structural regions of the RrQR enzyme. Therefore, the stereospecific catalytic activity of RrQR would be achieved by this novel substrate-binding mechanism (Figure 4d).

## Discussion

The structure of RrQR is the first structure of quinuclidinone reductase shown to catalyze the stereo-specific reduction of 3-quinuclidinone. The RrQR enzyme produces (*R*)-3-quinuclidinol with more than 99.9% enantiomeric excess (Uzura et al. 2009). The angles of the hydride transfer reaction (C---H---C) have been suggested to be rigidly restricted (Wu and Houk 1987a; Wu and Houk 1987b). However, the structural model indicates that RrQR recognizes the substrate within a narrow area (Figure 4). In agreement with this, the *K*_*m*_ value for 3-quinuclidinone (440 mM) is relatively high. Therefore, the RrQR enzyme would bind loosely to the substrate, but the substrate-binding site is sufficient to accommodate 3-quinuclidinone in one direction to produce the *R*-form of 3-quinuclidinol.

The substrate orientation is restricted by the circumstances of the catalytic site of RrQR. The substrate-binding pocket of RrQR is largely formed by the hydrophobic wall. The surface of the 3-quinuclidinone is divided into two parts, a hydrophilic face and hydrophobic face. Thus, the asymmetric reduction of 3-quinuclidinone is achieved by the ability of RrQR to discriminate the hydrophobic surface from the hydrophilic surface of 3-quinuclidinone. The hydrophobic residues of I167 and F212 function not only in the stereospecific substrate-binding but also in the substrate accommodation leading to the catalytic reaction (Table 2), and therefore we could not analyze the effect of the mutagenesis on the stereoselectivity. Several compounds, including 4-chloro-3-oxobutanoate and ketopantoyl lactone, are catalyzed by RrQR, but the optical purities of the products are reduced (Uzura et al. 2009). Therefore, the size and the chemical property of the catalytic pocket of RrQR is suitable for the binding of 3-quinuclidinone in the specific orientation.

Structural and mutation analyses of TR enzymes have indicated that the enzymes utilize electrostatic interactions between an amine group of the substrate and the carboxyl group of a Glu residue to determine the substrate-binding orientations (Nakajima et al. 1998; Nakajima et al. 1999). Similarly, levodione reductase also utilizes the electrostatic interaction between a positively charged surface of the substrate and a Glu residue to bind the substrate in the specific orientation (Sogabe et al. 2003). In contrast, the RrQR enzyme retains the stereospecific activity exhibited by the hydrophobic wall. Therefore, RrQR possesses a novel mechanism for the stereospecific reaction led by a characteristic interaction, in which the hydrophobicity of I167 and F212 plays a crucial role in the stereospecific reduction of 3-quinuclidinone.

In conclusion, we have described the structural analysis of an enzyme that catalyzes the stereospecific reduction of 3-quinuclidinone to (*R*)-3-quinuclidinol. The three-dimensional substrate-binding model and the mutation analysis indicate that the specific substrate-binding would be achieved through the interactions between hydrophobic residues and a hydrophobic substrate surface. The hydrophobic interactions would be crucial for the asymmetric reduction of 3-quinuclidinone. The reason for the low affinity for the substrate is that the compound 3-quinuclidinone is not the natural substrate for the enzyme. Therefore, it may be possible that tuning of the enzyme by genetic strategies and/or optimization of the reaction condition will yield an effective system for the expression of its maximum activity. The structural insights uncovered by this study will provide valuable information for investigations of enzymes that catalyze small cyclic molecules in specific orientations.

## Competing interests

The authors declare that they have no competing interests.

## Supplementary Material

Additional file 1 Supplementary data associated with this article can be found in the online version. Click here for file
