# Supplementary material for: Structural basis of stereospecific reduction by quinuclidinone reductase
Source: AMB Express. 2014 Feb 7;4:6. doi: 10.1186/2191-0855-4-6 (PMC3922912; doi:10.1186/2191-0855-4-6)
Supplement: Additional file 1 — Supplementary data associated with this article can be found in the online version. [file 2191-0855-4-6-S1.pdf]

AMB Express

Supplementary Material

Structural basis of stereospecific reduction by quinuclidinone reductase

Daijiro Takeshita,[1] Michihiko Kataoka,[2,3] Takuya Miyakawa,[1] Ken-ichi Miyazono,[1] Shoko Kumashiro,[2] Takahiro Nagai,[2] Nobuyuki Urano,[2,3] Atsuko Uzura,[4] Koji Nagata,[1] Sakayu Shimizu[2,5] and Masaru Tanokura[1]*

[1]Department of Applied Biological Chemistry, Graduate School of Agricultural and Life Sciences, University of Tokyo, 1-1-1 Yayoi, Bunkyo-ku, Tokyo 113-8657, Japan

[2]Division of Applied Life Sciences, Graduate School of Agriculture, Kyoto University, Kitashirakawa-Oiwakecho, Sakyo-ku, Kyoto 606-8502, Japan

[3]Division of Applied Life Sciences, Graduate School of Life and Environmental Sciences, Osaka Prefecture University, 1-1 Gakuencho, Naka-ku, Sakai, Osaka 599-8531, Japan

[4]Research and Development Center, Nagase & Co., Ltd., 2-2-3 Murotani, Nishi-ku, Kobe 651-2241, Japan

[5]Faculty of Bioenvironmental Science, Kyoto Gakuen University, Sogabe-cho, Kameoka 621-8555, Japan

*To whom correspondence should be addressed, at the above address. E-mail: amtanok@mail.ecc.u-tokyo.ac.jp; Phone: +1-3-5841-5165; FAX: +81-3-5841-8023

Table S1. Statistics of the data collection of the RrQR crystal in the resolution range 2.98 Å to 2.20 Å. The diffraction data up to 2.2 Å resolution were used for the structural refinement.

Resolution (Å)	R_{merge} (%) [a]	Redundancy	Completeness (%)
2.98 – 2.77	11.7	12.5	99.4
2.77 – 2.61	12.8	12.6	98.9
2.61 – 2.48	14.3	12.7	98.8
2.48 - 2.37	16.1	12.2	98.6
2.37 – 2.28	20.9	9.9	93.1
2.28 – 2.20	23.9	8.9	81.0

[a] $R_{merge} = \Sigma_{hkl} \Sigma_i |I_i(hkl) - \langle I(hkl) \rangle| / \Sigma_{hkl} \Sigma_i I_i(hkl)$, where $I_i(hkl)$ is the *i*-th intensity measurement of reflection *hkl*, including symmetry-related reflections, and $\langle I(hkl) \rangle$ is its average.

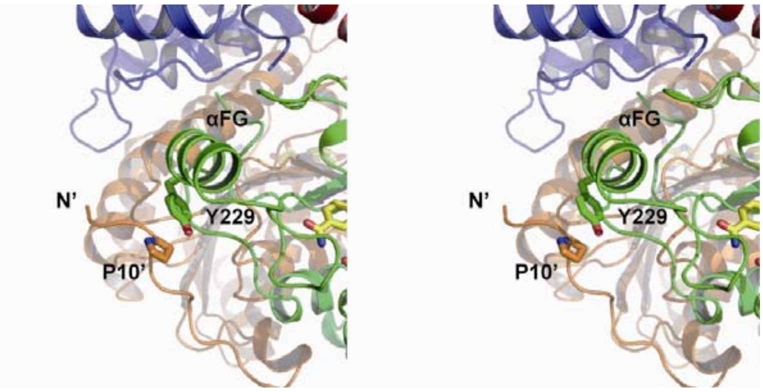

Fig. S1 Residues P10 and Y229 of a neighboring subunit shown by the stick model form a stacking interaction. Protomers in the tetramer are colored green, orange, blue, and red.

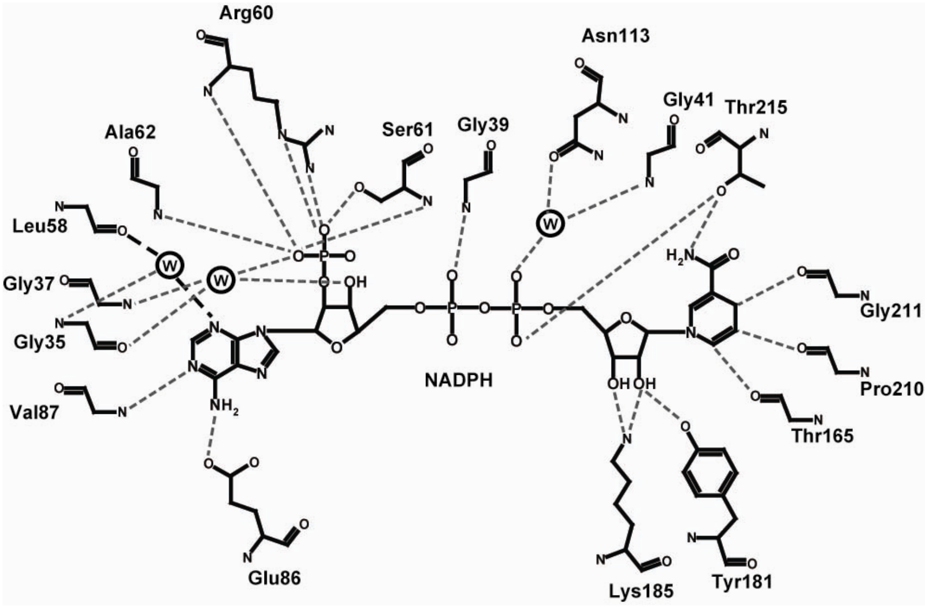

Fig. S2 The recognition of NADPH by RrQR. Hydrogen bonds are indicated by dotted lines. Water molecules involved in NADPH binding are shown.

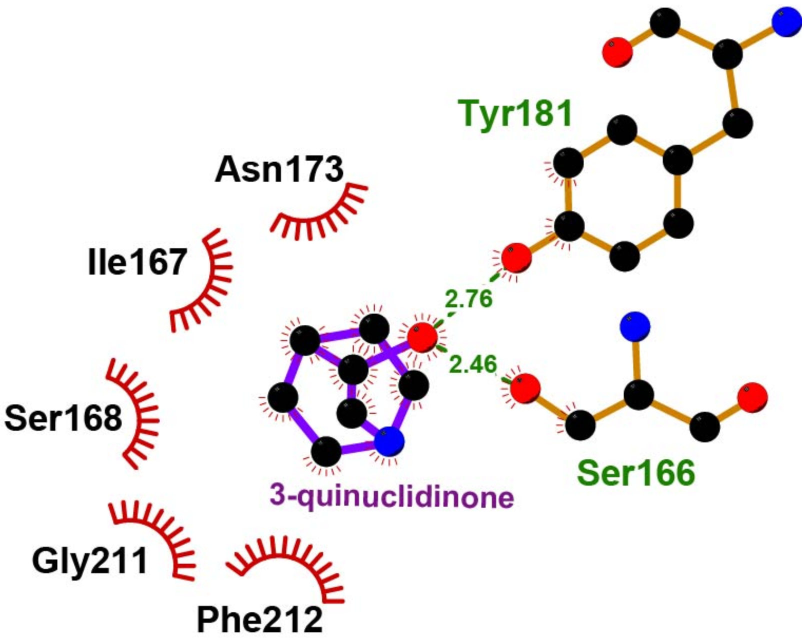

Fig. S3 Residues interacting with the substrate. The schematic was prepared by the program Ligplot (Laskowski et al. 2011) using the catalytic model in Fig. 4.
